# Demographic Profile of Benign and Malignant Oral Tumors in Central India: A Retrospective Comparative Study

**DOI:** 10.7759/cureus.25345

**Published:** 2022-05-26

**Authors:** Suhani Ghai, Yogesh Sharma

**Affiliations:** 1 Surgical Oncology, Dharamshila Narayana Superspeciality Hospital, New Delhi, IND; 2 Oral and Maxillofacial Surgery, Netaji Subhash Chandra Bose (NSCB) Medical College, Jabalpur, IND

**Keywords:** public health, tobacco, benign and malignant tumors, demographic profile, epidemiology

## Abstract

Background: Oral malignancy is endemic in India due to high addiction to tobacco and betel nuts. In addition, benign oral tumors are also very common in India. Studies comparing the demographic profile of benign and malignant oral tumors are scarce in India.

Methods: In this retrospective study, biopsy records of patients with solid tumors who presented to the Oral and Maxillofacial Surgery department from 2006 to 2018 were analyzed. The age and gender distribution of benign and malignant tumors were compared using Student's t-test and Fisher's exact test, respectively.

Results: Out of the 429 biopsies reported, non-neoplastic lesions, which included hyperplasias/dysplasia (107) and cystic lesions (113), were excluded, while neoplastic lesions (209) were included in our study. Out of these, 77 (37%) were malignant while 132 (63%) were benign tumors. Among the benign tumors, the most common were fibromas of various types (52/132, 39%) and odontogenic tumors (33/132, 25%); and among the malignant tumors squamous cell carcinoma was the most common (64/77, 83%). The mean age of patients with malignant tumors was significantly higher than benign tumors (51±14 versus 32±16 years; p<0.01). Alarmingly, 23% of malignant tumors were seen in patients ≤40 years of age.

Conclusion: In central India, 37% of all neoplasms of the oral cavity are malignant 63% are benign. The average age of presentation of malignant oral tumors is 51 years, and almost one-fourth of all oral malignancies occur in patients below 40 years of age. The high frequency of younger patients developing oral cancer calls for urgent measures to spread awareness about oral cancer and its causative factors in India.

## Introduction

The abnormal growths that can be seen in the oral cavity are diverse and comprise a broad spectrum of either benign or malignant lesions [1]. Poor oral hygiene, tobacco and betel nut chewing, smoking, removable dentures, malposition, and mechanical irritation predispose to reactive lesions as well as tumor development. The diagnosis of oral cavity lesions is established from clinical and radiological features; however, the final diagnosis is based on a histopathological examination of the lesion.

A wide variety of benign tumors are present in the oral cavity. They are generally slow-growing, noninvasive, with scant mitosis and high cellular differentiation. Recurrence is rare, and they never metastasize. Although benign oral cavity tumors are not life-threatening, they can result in extensive loss of soft tissue and/or bone. The most common benign tumors are fibroma and odontogenic tumors. Clinical differentiation between benign and malignant tumors is difficult, hence histopathological confirmation is required. Furthermore, many patients are subject to the threat of recurrence, multiple surgical procedures, and the possibility of malignant degeneration [2,3].

Oral cancer is known to be a heterogeneous group of cancer and arises from different parts of the oral cavity. It has different predisposing factors, prevalence, and treatment outcomes. It is the sixth most common cancer reported globally and has an annual incidence of over 300,000 cases, out of which 62% arise in developing countries [4]. In the U.S. population, oral cavity cancer represents only about 3% of the malignancies but it accounts for over 30% of all the cancers in India [4]. There is a significant difference in the incidence of oral cancer in different regions of the world. The age-adjusted rates vary from over 20 per 100,000 population in India to 10 per 100,000 in the U.S.A and less than two per 100,000 population in the Middle East [4,5]. The variation in the incidence and pattern of oral cancer can be attributed to the regional differences in the prevalence of risk factors. Similarly, a wide variation is seen in demographic characteristics of benign oral tumors in different geographical regions of the world.

Oral malignancy is endemic in India due to high addiction to tobacco (especially in males) and betel nut (especially in females) [6]. Benign tumors (like fibromas and odontogenic tumors) which have similar risk factors as malignant lesions [7] are also significant in India along with malignant tumors. In India, a broad spectrum of incidence of these tumors makes a regional epidemiological study necessary. Descriptive and detailed oral tumor data for each specific geographic area is important for many reasons. It helps in understanding the extent of the problem, determining the groups within the population that are at the highest and the lowest risk, and relating the burden of the oral tumor to that of other tumors to evaluate and estimate the allocation of resources for research, prevention, treatment, and support services [8-10].

This retrospective study aimed to compare the demographic profile of benign and malignant oral tumors in Central India. Very few other studies have been conducted in different parts of India on the epidemiology of oral cancer and benign tumors and to the best of our knowledge, no study has been conducted on the Central Indian population. 

## Materials and methods

Patients

This was a retrospective study and the biopsy records of patients presenting with solid tumors to the Oral and Maxillofacial Surgery (OMFS) department of People's Dental Academy, Bhopal, India, from August 2006 to June 2018 were analyzed.

Inclusion Criteria

Patients of any age or gender with a suspected diagnosis of solid oral tumors were referred to the OMFS department for diagnosis and management. Only those patients were included whose records were complete. 

Exclusion Criteria

Oral cystic lesions; focal fibrous hyperplasias; dysplasias; verrucous leukoplakias and hyperplasias; inflammatory lesions; oral sub-mucous fibrosis; and sialolithiasis.

Histopathology

A scalpel biopsy, for both incisional and excisional procedures, was obtained in all the cases. Histopathological diagnoses were classified into benign tumors (Fibroma, Ameloblastoma, Giant cell granuloma, Lipoma, Peripheral ossifying fibroma, Pleomorphic adenoma, and others) and malignant tumors (squamous cell carcinoma, verrucous carcinoma, mucoepidermoid carcinoma, fibrosarcoma, papillary cystadenocarcinoma, and others). Histopathological diagnoses were classified according to the WHO classification of head and neck tumors [11]. Immunohistochemical stains were used for diagnosing those tumors that could not be evaluated via common or routine stains such as hematoxylin and eosin. Our center being a teaching hospital, each histopathology slide was examined by a trained senior resident as well as a teaching faculty. Any discordance in diagnosis was resolved with discussion or by taking the opinion of additional faculty members of the department. For the purpose of this study, the histopathological diagnosis given in the report was used and histopathology slides were not reviewed again. However, if the report was ambiguous or with an unclear diagnosis, then the slides were reviewed again.

Comparisons and statistical methods

The age and gender distribution of benign and malignant tumors were compared using Student's t-test and Fisher's exact test, respectively. A p-value below 0.05 was considered significant. Statistical analysis was performed using the Statistical Package for Social Sciences (SPSS) version 22.0 (SPSS Inc., Chicago, IL).

Ethical statement

This being a retrospective study, an Institutional Ethics Committee clearance was not required. As per the standard protocol of our department, informed consent was obtained from each patient prior to the biopsy procedure. The informed consent document not only included consent for the procedure but also consent for using the data for any study purpose. All procedures performed in this study were in accordance with the ethical standards of the institutional and national research committee and conformed to the 1964 Helsinki declaration and its later amendments.

## Results

Patients

**Figure 1:**
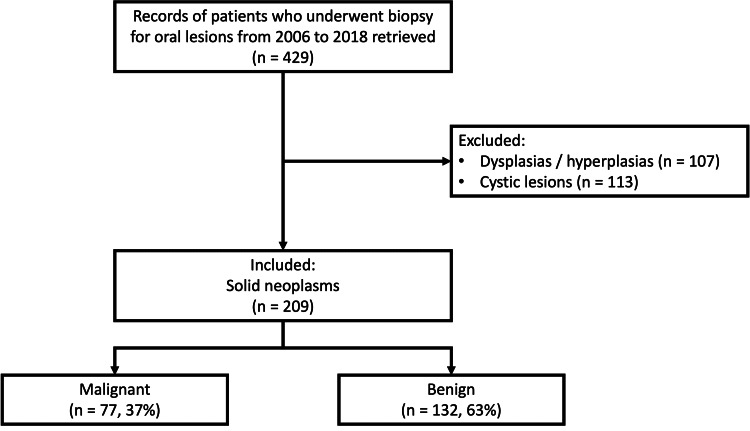
Flow of data collection.

Biopsy records were retrieved from August 2006 to June 2018. A total of 464 biopsy records were analyzed. Of these 255 patients were excluded due to following reasons: oral cystic lesions (n=113); focal fibrous hyperplasias (n=67); dysplasias (n=53); verrucous leukoplakia and hyperplasia (n=8); inflammatory lesions (n=7); oral sub-mucous fibrosis (n=5); and sialolithiasis (n=2). Two hundred and nine remaining biopsies were solid tumors and were therefore included in the study (Figure 1).

Histopathological diagnosis

Of the 209 included patients, 77 (37%) were malignant tumors while 132 (63%) were benign. Fibromas were the most common (52/132, 39%) among benign tumors; and squamous cell carcinoma was the most common (64/77, 83%) among malignant tumors. Table 1 gives the diagnosis of all the tumors.

**Table 1:** Histopathological diagnosis of the tumors.

Nature	Histological Diagnosis	n, ratio, %
Benign (n=132)	Fibromas of various types	52/132, 39%
	Fibroma	35
	Peripheral ossifying fibroma	8
	Juvenile ossifying fibroma	3
	Cemento-ossifying fibroma	3
	Peripheral cementifying fibroma	2
	Neurofibroma	1
	Odontogenic tumor	33/132, 25%
	Ameloblastoma	21
	Complex odontoma	6
	Adenomatoid odontogenic tumor	4
	Calcifying odontogenic cyst	1
	Ameloblastic fibro odontome	1
	Lipoma (including fibrolipoma)	10/132, 8%
	Giant cell granuloma	9/132, 7%
	Pleomorphic adenoma	8/132, 6%
	Vascular lesion	7/132, 5%
	Papilloma	6/132, 4%
	Others	7/132, 5%
Malignant (n=77)	Squamous cell carcinoma	64/77, 83%
	Verrucous carcinoma	7/77, 9%
	Mucoepidermoid Carcinoma	4/77, 5%
	Fibrosarcoma	1/77, 1%
	Papillary cyst adenocarcinoma	1/77, 1%

Age comparison between benign and malignant tumors

**Figure 2:**
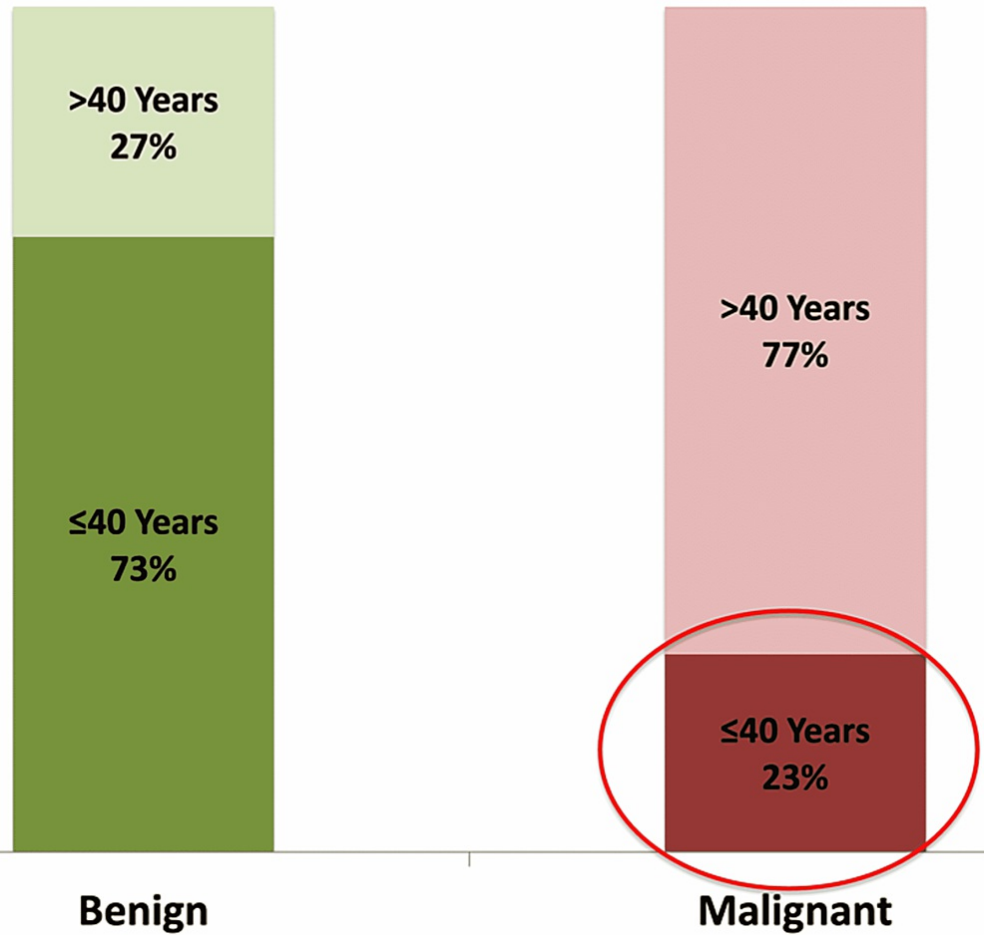
Age comparison of benign and malignant tumors.

The mean age of patients with malignant tumors was significantly higher (51±14 versus 31±16 years; p<0.01) as compared to benign tumors. Alarmingly, 23% of oral malignancies were seen in patients ≤40 years of age (Figure 2).

Gender comparison between benign and malignant tumors

**Figure 3:**
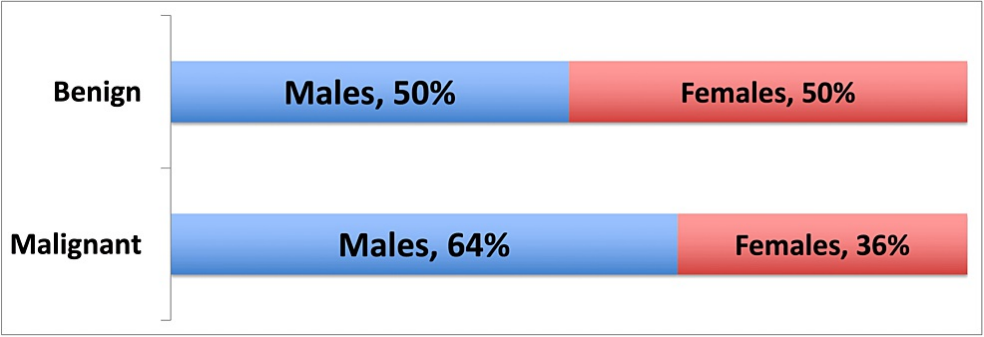
Gender comparison on benign and malignant tumors.

The male:female ratio of benign tumors was 1:1 and malignant tumors were 1.8:1. Although malignant tumors were slightly more in the male gender than female, this difference was not statistically significant (49/98 [50%] versus 28/76 [37%]; p=0.092) (Figure 3).

## Discussion

Our study showed that in Central India, 37% of all solid oral tumors are malignant, with the most common malignancy being squamous cell carcinoma. The mean age of presentation of these malignant tumors is 51 years, and about one-fourth of the malignancies are present in patients younger than 40 years of age. Among benign oral tumors, fibromas of various types and odontogenic tumors are the most common types. The age of presentation of benign tumors is lower than that of malignant tumors, usually in the third decade of life. The results of gender distribution showed that there was no statistically significant difference in the gender distribution between malignant and benign tumors.

The densely populated Central India has a population of more than 100 million, and to the best of our knowledge, ours is the first study from Central India which compares the demographic profile of benign and malignant oral tumors. We found an alarmingly high proportion of oral neoplasm to be malignant (37%). From North India, a similar study reported that of the 1,151 oral biopsies, 32% were malignant [12]. Another South Indian study found malignant neoplasms to be 27% out of 60 neoplastic lesions biopsied from gingiva [13]. A smaller South Indian study found malignancies to be 61% of 75 biopsied neoplasms from gingiva [14]. In a study from the Western Indian state of Gujarat, out of 28 gingival neoplastic lesions, only 7% were malignant [15]. Thus, although there is a wide variation in the frequency of malignancies in patients with oral neoplasms in India depending on geographic location; the proportion of malignancies in the Central Indian region seems to be the highest. The established risk factors for oral cancer in the Central Indian population include tobacco abuse in various forms (such as gutka, zarda, mawa, kharra, khaini, cigarettes, bidi, hookah, etc.); the chewing of betel quid; alcohol abuse; and the presence of premalignant disorders. Other contributory or predisposing factors include viral infections, particularly by HPVs with high oncogenic potential [16]. We found that squamous cell carcinoma was the most common malignant tumor comprising 83% of all oral malignancies. Verrucous carcinoma was found to be the second most common malignant tumor in our study. Our study is in agreement with most other Indian studies which also report squamous cell carcinomas as the most frequent oral malignant lesions [12-15].

Benign tumors comprised 63% of all solid oral neoplasms. Similar to other studies from India, our study also found that fibromas of various types were the most common benign tumors comprising 39% of all benign tumors, followed by odontogenic tumors at 25%. A study conducted in the Western Indian population found fibroma (31%) and fibro-lipoma (27%) to be the most common benign oral tumor, followed by peripheral ossifying fibroma (23%) and peripheral giant cell granuloma (11%) [15]. Fibroblast and collagen fiber proliferation results in the formation of fibroma [17]. Most cases of fibroma occur due to the progression of fibrous hyperplasia as a result of chronic irritation and true fibroma is rare [17,18]. Peripheral ossifying fibroma is a fibroma of the gingiva in which there are areas of calcification or ossification [19]. As a result of its clinical and histopathological resemblance, some peripheral ossifying fibromas are thought to develop from pyogenic granuloma which undergoes fibrous maturation and subsequent calcification [19]. Odontogenic tumors arise from the tissues of tooth formation and form a small but diverse group of lesions. In our study, among the odontogenic tumors, Ameloblastoma was the most common (21/33, 64%). Other Indian series on odontogenic tumors have also reported ameloblastoma to be the most frequent odontogenic tumor [20,21].

On analyzing the gender distribution between the malignant and benign oral neoplasms our study found that for benign tumors, gender predilection was same (1:1) but malignant tumors had slightly more predilection for males (1.8:1). Manjunatha et al. [15] found in their study that out of the total benign neoplasms studied, 69% were found in females and 31% were found in males, hence showing female predilection. Torres-Domingo et al. [17] also found greater prevalence in females than males (2:1) in their study of benign lesions of oral mucosa. Benign lesions like fibromas, pyogenic granulomas, and giant cell granulomas had greater predilection in women [17]. Other studies conducted for malignant tumors found greater male to female ratio. Sharma et al. [8] from Western UP and Ganesh et al. [22] from Tamil Nadu, conducted studies on demographic characteristics of oral cancer and noted that squamous cell carcinoma was more common in males with a ratio of 2.2:1 and 2.1:1 respectively. While Shenoi et al. from Nagpur reported even higher male-to-female distribution (4.18:1) [23]. This difference in distribution can be attributed to increased addiction to tobacco and betel nut consumption in males as compared to females. Also, in India, tobacco and alcohol consumption is considered socially unacceptable for females but slowly this trend is changing, and incidence of oral malignancies is on rise among females as well.

Our study found that the age of presentation of benign neoplasm was between second and fourth decades of life with mean age of presentation being 32±16 years and malignant neoplasm presented between third and sixth decades with mean age of presentation being 51±14 years. Shamim et al. [13] found a maximum incidence of benign tumors between the age group of 10-19 years (18.2%) followed by the age group of 40-49 years (15.9%) and 50-59 years (15.9%). They found maximum number of oral carcinoma patients were between the ages of 40-49 years. Another study found benign lesions among subjects in the age range of 10 to 39 years, and malignant lesions among subjects in the age range of 50 to 69 years [14]. A study conducted on population of western UP found peak incidence of oral cancer in fourth and fifth decades [8]. Thus many studies indicate that the mean age of presentation for malignant tumors is higher than benign tumors. An alarming finding in our study was that 23% of malignant tumors were found to be occurring in a younger age group (<40 years), which call for urgent measures to address tobacco and betal nut consumption habits in population in their reproductive age group. Some recent studies conducted in Western countries show that the oral malignancy incidence is increasing in young male patients (<40 years of age) [8,24,25].

Our study had several limitations. First, this being a retrospective study from the available records, we did not have full clinical details of the patients such as clinical presentation, previous treatment history and associated comorbidities. Second, for the same reason we also did not have the full details of addiction history (betel nut, tobacco, smoking, alcohol, etc.) of the patients. Third, the human papilloma virus (HPV) status of the patient was also not assessed. Fourth, since many patients obtained the subsequent treatment of their lesion at other centers, we do not have the treatment and follow-up details of the patients. Further larger multicentric studies on epidemiology of oral tumors should be conducted in India, especially for mapping the geographic distribution of malignant and benign tumors, and for spreading awareness among the dental fraternity and general public in order to reduce the incidence of oral cancer in India.

The most important clinical significance of our article is to spread the awareness among the dentists and otolaryngologists of India that even in patients younger than 40 years of age, malignant neoplasms are common, and if any growth is detected a high index of suspicion for malignancy should be kept.

## Conclusions

In conclusion, in central India, which is endemic for oral cancer, 37% of all neoplasms of the oral cavity are malignant and 63% are benign. The average age of presentation of malignant neoplasms is 51 years, and almost one-fourth of malignant tumors occur in patients younger than 40 years of age. The high frequency of younger patients developing oral cancer calls for urgent measures to spread awareness about oral cancer and its causative factors in India.
